# Refining Tumor Treatment in Sinonasal Cancer Using Delta Radiomics of Multi-Parametric MRI after the First Cycle of Induction Chemotherapy

**DOI:** 10.3390/jimaging8020046

**Published:** 2022-02-15

**Authors:** Valentina D. A. Corino, Marco Bologna, Giuseppina Calareso, Carlo Resteghini, Silvana Sdao, Ester Orlandi, Lisa Licitra, Luca Mainardi, Paolo Bossi

**Affiliations:** 1 Department of Electronics, Information and Bioengineering, Politecnico di Milano, 20133 Milan, Italy; marco.bologna@polimi.it (M.B.); luca.mainardi@polimi.it (L.M.); 2 Radiology Department, Fondazione IRCCS Istituto Nazionale dei Tumori di Milano, 20133 Milan, Italy; giuseppina.calareso@istitutotumori.mi.it (G.C.); silvana.sdao@istitutotumori.mi.it (S.S.); 3 Head and Neck Medical Oncology Department, Fondazione IRCCS Istituto Nazionale dei Tumori di Milano, 20133 Milan, Italy; carlo.resteghini@istitutotumori.mi.it (C.R.); ester.orlandi@istitutotumori.mi.it (E.O.); lisa.licitra@istitutotumori.mi.it (L.L.); 4 Department of Oncology and Hemato-Oncology, University of Milan, 20122 Milan, Italy; 5 Department of Medical and Surgical Specialties, Radiological Sciences and Public Health, University of Brescia, 25123 Brescia, Italy; paolo.bossi@unibs.it

**Keywords:** radiomics, magnetic resonance imaging, sinonasal cancer, machine learning, delta radiomics

## Abstract

Background: Response to induction chemotherapy (IC) has been predicted in patients with sinonasal cancer using early delta radiomics obtained from T1- and T2-weighted images and apparent diffusion coefficient (ADC) maps, comparing results with early radiological evaluation by RECIST. Methods: Fifty patients were included in the study. For each image (at baseline and after the first IC cycle), 536 radiomic features were extracted as follows: semi-supervised principal component analysis components, explaining 97% of the variance, were used together with a support vector machine (SVM) to develop a radiomic signature. One signature was developed for each sequence (T1-, T2-weighted and ADC). A multiagent decision-making algorithm was used to merge multiple signatures into one score. Results: The area under the curve (AUC) for mono-modality signatures was 0.79 (CI: 0.65–0.88), 0.76 (CI: 0.62–0.87) and 0.93 (CI: 0.75–1) using T1-, T2-weighted and ADC images, respectively. The fuse signature improved the AUC when an ADC-based signature was added. Radiological prediction using RECIST criteria reached an accuracy of 0.78. Conclusions: These results suggest the importance of early delta radiomics and of ADC maps to predict the response to IC in sinonasal cancers.

## 1. Introduction

Sinonasal cancers (SNCs) represent a group of rare and heterogenous tumors arising in the nasal cavity and sinuses. Their incidence is less than 1 case per 100,000 individuals; SNCs account for less than 15% of head and neck cancers [1]. Survival depends on the type of histology and on the disease extent. Therapeutic opportunities are mainly represented by surgery and radiation therapy, with chemotherapy being useful in selected and more responsive histotypes. Therefore, SNCs often represent a challenge, both in diagnostic and therapeutic choices. It has been suggested that multimodality treatment improves overall survival [2,3] and, in particular, induction chemotherapy (IC) may have a favorable impact on outcomes [3,4]. In analogy with other subsites in the head and neck area, IC has been considered a possible way to improve organ preservation in the locally advanced paranasal sinus and nasal cavity [5]. The overall response to IC has been shown to be a strong prognostic factor for OS [2,4] for overall survival. However, unanswered questions regarding predictive markers of response to IC remain, specifically, which patients with SNCs could benefit from IC? Can they be identified using pretreatment or early treatment information?

Recently, radiomics, a method that extracts a large number of image characteristics, has been applied to oncology to predict the response to treatment as well as overall survival. In particular, T1-weighted, T2-weighted and diffusion-weighted images (DWI) have already been exploited in radiomics’ studies of head and neck cancer, for tumor diagnosis [6], stratification [7] and prognosis [8]. Using radiomics on MRI images may be a powerful tool for helping clinicians to characterize SNCs and to choose the best, patient-tailored clinical treatment path. Recently, radiomics-based predictive models of response to IC in SNCs have been tested, highlighting the relevance of baseline apparent diffusion coefficient (ADC)-based radiomics for response prediction [9], with an area under the curve (AUC) of 0.78. In that study, radiomic features were considered only at baseline imaging. However, in the case of longitudinal analysis, radiomic features can be computed at multiple time points, and in those cases, the delta radiomic features, that is, the difference in radiomic features between two time points, can be computed as well. Delta-radiomics can provide abundant information to identify, quantify and potentially predict therapy-induced changes over the course of treatment and has been successfully applied to other tumors [10].

The aim of this study was to use delta radiomics (with the first early evaluation performed three weeks after starting IC therapy) to predict response to IC in patients with SNCs, as early identification of the patients benefitting from IC could be crucial in defining the therapeutic journey for SNCs. Moreover, early (i.e., three weeks after starting IC therapy) radiological evaluation by RECIST (Response Evaluation Criteria in Solid Tumors (RECIST)) 1.1 [11] was tested as a predictor of IC response and compared to delta-radiomics prediction.

## 2. Materials and Methods

### 2.1. Study Population

The present study is based on patient data collected in two studies, two phase II Multicentric Clinical Trials. The aim of these trials was to evaluate the impact of tailored IC, followed by locoregional treatments in resectable (SINTART 1) and unresectable (SINTART 2) epithelial SNCs (ClinicalTrials.gov identifiers: NCT02099175 and NCT02099188). The ethical committee of the coordinating and participating centers approved these studies, and all patients provided written informed consent. All patient data were anonymized prior to the analysis.

After a clinical evaluation by a multidisciplinary team (surgeons, medical and radiation oncologists), head and neck MRI images were acquired and the therapeutic strategy was decided, assigning the patient to SINTART 1/SINTART 2 if surgical resection was/was not feasible in terms of obtaining radical resections. The patients in both trials underwent histology-driven IC for up to five cycles, 3 weeks ± 3 days apart from each other, according to tolerability and tumor response. The probability of response to IC is much higher in the first cycles of therapy; therefore, in the clinical protocol, IC was administered for maximum five cycles and only to patients achieving a tumor size reduction of at least 10% after the first cycle and 50% after the 3rd cycle. This was conducted to maximize the possibility of offering effective treatment without causing unnecessary toxicities. After the IC phase, patients in Study 1 underwent surgery in cases of residual disease greater than 20% with respect to the initial assessment. Both groups were then treated with radiation therapy, with or without concurrent chemotherapy, according to pathologic report findings or tumor response to IC. The IC response was evaluated using RECIST at the end of the induction treatment period (thus it was a radiological response). Fifty-three patients enrolled in the clinical trials were considered. Three patients were excluded because IC response data were not available; thus 50 patients were included in this study. Most of the patients were men (*n* = 41), and their age was 54 ± 12 years, range 34–78 years. Other clinical characteristics of the two populations divided by responses are reported in Table 1.

Patients were categorized into two groups according to IC response (defined according to RECIST 1.1, at the end of the treatment): (i) NR (non-responders) patients, including patients with progressive (PD) or stable (SD) disease at the end of the IC treatment; (ii) R (responders) patients, including patients with partial (PR) or complete (CR) remission. Both the groups included 25 patients.

### 2.2. Image Acquisition

T1-weighted images were acquired for 49 patients, T2-weighted images for all 50 patients, and DWI was performed only in 34 patients. Thirty-three patients had all three image types. T1-weighted and T2-weighted images were acquired using turbo-spin echo pulse sequences, while DWI were acquired using echo planar imaging. Image acquisition parameters are shown in Supplementary Table S1. Images were evaluated for response to IC according to RECIST 1.1 by an expert radiologist (GC, 10-year experience) after the first cycle (early RECIST) and at the end of the treatment. Example images of a patient at baseline and three weeks after starting IC therapy are shown in Figure S1.

### 2.3. Image Preprocessing and Segmentation

For each DWI series, the ADC was computed as the slope of the linear regression of the logarithm of the DWI exponential signal decay on the b values [12]. The calculations were performed voxel-wise using the open-source library ITK (version 4.8).

The intensity values between the minimum and 98th percentile of the voxel intensity of the images were mapped on a fixed range of intensities ([0–5000]) for T1-weighted and T2-weighted images. No intensity normalization was performed on ADC maps [13,14,15].

An expert radiologist (GC, 10-year experience) segmented the region of interest (ROI) on the T2-weighted images; ROIs were confirmed by another radiologist (SS) and disagreements were resolved by consensus to decrease observer bias.

T2-weighted images were chosen as a reference for the segmentation because they show the highest contrast between tumors and surrounding tissues, and also because they were available for every patient in this study. The ROI delineation was performed using Philips Intellispace software (Philips Healthcare, Best, The Netherlands). The segmented ROI was then mapped to T1-weighted and ADC images to compute the features of those images. The segmentation was performed slice-by-slice by the radiologist, but the final result was a 3D ROI that was used as a mask to extract the radiomic features.

Voxel-size resampling to an isotropic resolution of 2 mm was performed using B-spline interpolation.

### 2.4. Radiomic Features Extraction

The extraction of radiomic features was performed using Pyradiomics 3.0 [16]. A total of the 536 features were extracted for each image type. Since features were extracted with Pyradiomics, they were compatible with the image biomarker standardization initiative (IBSI) [17]. The extracted features belonged to different categories: shape and size (SS, 14 features), first-order statistics (FOS, 18 features) and textural (40 features). Textural features were computed using the grey level co-occurrence matrix (GLCM) and the grey level run length matrix (GLRLM). The FOS and textural features were also computed for the eight images obtained by the first-level wavelet decomposition of the MRI volume (wavelet, for a total of 464 features). The full list of radiomic features is available in the Pyradiomics documentation [17]. Thirty-two bins were used for gray-level discretization.

Radiomic features were computed for each image type (T1-weighted images, T2-weighted images and ADC maps), at baseline and after the first IC cycle. For each feature, early delta radiomics, defined as the difference between the value after the first IC cycle and that at baseline, was computed and used as a feature for the following classification.

### 2.5. Features Selection and Classification

A first selection was based on feature stability, as found in [18]. Briefly, features robust to changes in TR, TE, pixel spacing and slice thickness were identified, reducing the number of features to 279 and 387 for T1-weighted and T2-weighted MRI images, respectively. For the ADC maps, features that were stable for both T1-weighted and T2-weighted MRI images were selected, thus reducing the number of ADC features to 256 [9].

Because the values of radiomic features may differ by order of magnitude, Z-score normalization was applied to transform each feature distribution so that it had zero mean and unitary standard deviation.

The quality of each model was assessed through a train-validation-test split (55% of the data were used as the training set, 25% as the validation set, and 20% as the test set), repeated 100 times (bootstrapping). The training and validation sets were used to choose the best model, and the test set was used to test the model on the never-seen data. The data split was stratified by outcome (the proportion of responders and non-responders was the same in all the three sets).

In each iteration, a semi-supervised form of principal component analysis (PCA) was performed [19]. Briefly, PCA maps the original features in a different space such that each eigenvector (each principal component) is independent, thus eliminating the well-known problem of feature redundancy [20]. Instead of performing PCA on all features, semi-supervised PCA estimates the principal components on the features selected to be associated with the patient’s response (*p* < 0.05, Wilcoxon test, responders vs. non-responders). In this study, the principal components that were necessary to explain 97% of the total variance were selected. The transformation matrix computed by PCA on the training set was stored for application to the validation set. Finally, a sequential forward floating search (SFFS) algorithm was used to identify the optimal combination of principal components (on the validation set). Briefly, the SFFS algorithm [21], starting from an empty set of features, adds the principal component that maximizes the accuracy. In the case of multimodal imaging, PCA was performed after joining the feature sets from the different MRI sequences.

After feature selection, three mono-modality predictive models were developed, one for each image type (T1-weighted, T2-weighted and ADC), using the difference in the feature value after the first IC cycle and baseline, thus including pretreatment and early evaluation after the first IC cycle information. Not all patients had all the image types before treatment and after the first IC cycle; thus, different numbers of patients were considered in the different predictive models: 48 (25 responders), 49 (25 responders) and 28 (15 responders) patients for T1-weighted, T2-weighted and ADC, respectively.

Accuracy (the ratio between the correctly labeled results and the total number of cases), true positive rate (TPR, the ratio between the true positive cases and all the positive cases), true negative rate (TNR, the ratio between the true negative cases and all the negative cases) and the area under the curve (AUC) were computed to evaluate the model. The accuracy, TPR and TNR were averaged over the total number of repetitions. The online statistical tool Vassarstats (Vassar College, Poughkeepsie, NY, USA) was used to compare AUCs.

The classification algorithm used was a support vector machine trained on the training set and evaluated on the validation set to select the best number of principal components to use.

Each model provided a signature for each patient. From the mono-modality signatures, a distributed multi-agent decision-making algorithm [22] is used to fuse the signatures. In this study, a global prediction was obtained by averaging all available predictions. Global prediction overcomes the problem of missing signatures in ADC maps for some patients.

The classification based on delta radiomics was compared with that obtained at baseline, using both mono-modality and multi-modality models.

The entire workflow was implemented in MATLAB 2018b (MathWorks, Natick, MA, USA).

### 2.6. Comparison with Early Radiological Evaluation

Radiological evaluation by early RECIST was used as a predictor of IC response and performance metrics were used to evaluate it and compare it to the radiomic classification.

### 2.7. Comparison with Delta-Volume Model

To show that the radiomic signature provides larger predictive power than the simple computation of a tumor delta-volume value, a model based only on delta-volume was trained, and its performance metrics were compared to those of the radiomic signature.

## 3. Results

### 3.1. Radiomics Models

Among the 50 patients that were analyzed, the median number of IC cycles was 3 (interquartile range: 1–5). The two response groups were homogeneous, with 25 responders (6 CR and 19 PR) and 25 non-responders (1 PD and 24 SD).

**Figure 1:**
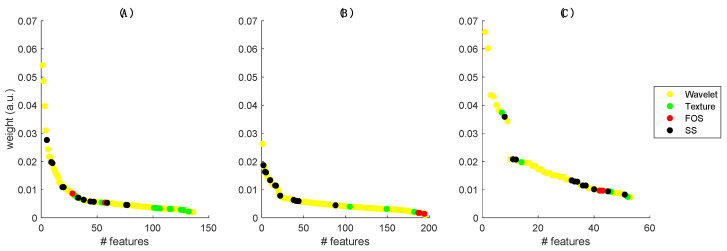
Relative weight of the various features in the principal components in: (**A**) T1-weighted, (**B**) T2-weighted and (**C**) ADC maps.

To understand the importance of the different features on the principal components selected for delta-radiomics-based classification, Figure 1 shows the feature relative weights highlighting the class of belonging (SS, texture, FOS and wavelets). The number of features for the different modalities (x-axis) is different because the semi-supervised PCA reduces the total number of features, preselecting only those correlated to the output. In T1- and T2-weighted images, it can be observed that higher weights are for features ranging to wavelets and SS, whereas in ADC maps, texture features are also relevant and the FOS group has very low weight in all modalities. The numbers of principal components necessary to obtain the 97% of the variance were 17, 16 and 9 for T1-, T2-weighted and ADC maps, respectively.

Table 2 shows the performance for the delta radiomics mono-modality classifiers, in terms of accuracy, true positive and negative rates and accuracy for the validation and test sets. The model with the best accuracy is the one based on the ADC maps (0.89 ± 0.13 in the validation set and 0.87 ± 0.13 in the test set), and the same is true when considering TPR (validation: 0.89 ± 0.19; test: 0.86 ± 0.19) and TNR (validation: 0.90 ± 0.18; test: 0.89 ± 0.20). Models based solely on T1- and T2-weighted images had comparable results for all the three metrics. When comparing the results of the radiomic models with those obtained by the baseline radiological examination, it is possible to see that radiomic models are much more sensitive to responders (higher TPR) but are less sensitive to non-responders (lower TNR), and it is also possible to see that the ADC-based model has a better accuracy. When comparing the results of the radiomic models with those obtained using only the volume, it can be noted that radiomic models have better accuracy and TPR, while having comparable or slightly lower TNR.

**Figure 2:**
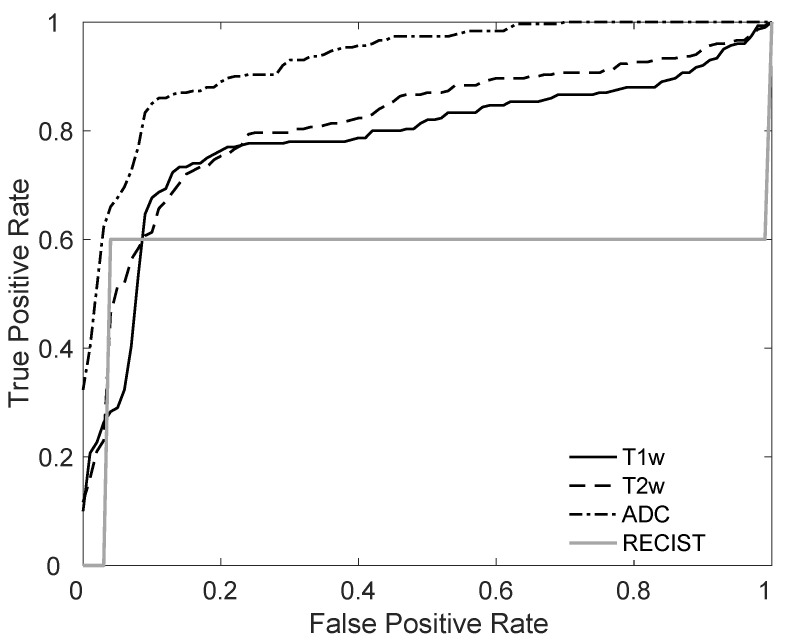
Receiver operating characteristics (ROC) curves for the mono-modality delta-radiomic predictive models and the RECIST-based model. The corresponding AUCs are 0.79 for T1w, 0.76 for T2w, 0.93 for ADC and 0.76 for RECIST.

Table 3 reports the mean AUCs based on the median signature for each patient, obtained by the test set. For the mono-modality classifiers, the model based on ADC maps showed the best AUC (*p* < 0.05, when compared with T1- and T2-weighted AUCs). When combining the signature, it can be noted that adding the ADC map-based signature increases the corresponding AUC, but never higher than the ADC maps alone (no significant difference can be found when comparing the fused signature AUC to the AUC of ADC maps). AUC values using only baseline features were much lower than the delta radiomics AUCs for the models used. It is worth noting that at baseline, fusing signatures of the three mono-modality models increased AUC compared to ADC maps only (AUC 0.83 and 0.79, respectively, even if not statistically significant). When comparing AUCs for delta radiomics model and baseline model, a statistically significant improvement (*p* < 0.05) can be observed for T2w and ADC. Figure 2 shows the receiver operating characteristics (ROC) curves of the mono-modality radiomic models, highlighting the best performance of the ADC-based model.

### 3.2. Comparison with Early Radiological Evaluation

**Figure 3:**
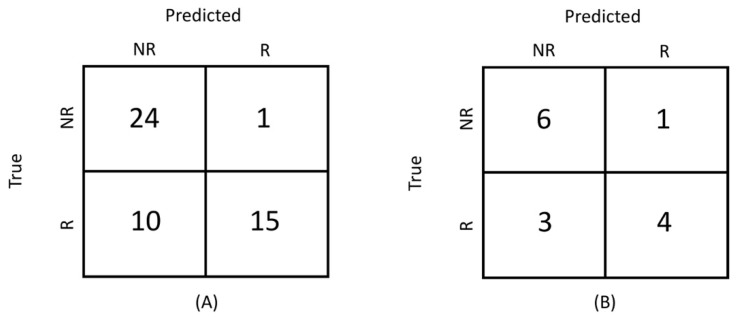
Average confusion matrix: (**A**) Performance of the radiological evaluation obtained using the early radiological evaluation as predictor. (**B**) Performance of the volume-based model. R = responders, NR = non-responders.

Figure 3A shows the performance (through the confusion matrix) of the radiological evaluation obtained using the early RECIST as a predictor for all patients (as no training was needed). It can be noted that early RECIST can predict non-responders very well, whereas 40% of the responders were not correctly classified, that is, 10 of the 25 patients, who did not respond after the first IC cycle responded after the complete treatment period. This corresponds to a very high TNR (0.96), but a much lower TPR (0.60), as reported in Table 2. The ROC curve using the early RECIST is shown in Figure 2.

### 3.3. Correlation with Delta-Volume

The model based on delta-volume only has lower performance compared to the best radiomic signature (ADC-based), reaching an AUC of 0.73 (0.70–0.75). The average confusion matrix on the test set is shown in Figure 3B, corresponding to a low TPR (0.56 ± 0.16) and a high TNR (0.89 ± 0.12), as shown in Table 2.

## 4. Discussion

The main finding of this study is that the prediction of the response to IC treatment through delta MRI-based radiomics is possible, reaching a minimum AUC of 0.76 when using only T2-weighted images and a maximum of 0.93 using the ADC maps in the test set. A median signature was obtained for all patients for the mono-modality analysis as well as a fused signature averaging the mono-modality signatures. When fusing signatures, adding information from the ADC maps improves the AUC values of T1- and T2-weighted images. This result confirms that ADC maps provide the most meaningful information for a predictive model of response to IC, as was found when analyzing only baseline images [9] and has been reported in other studies [8].

The second finding is that, compared to radiomics computed before treatment, delta radiomics (computed as the difference between radiomics after the first cycle and radiomics at baseline) largely improves the AUCs. In fact, AUC values increased from a maximum of 0.83 at baseline (obtained fusing signatures from the three modalities) to a maximum of 0.93 using delta radiomics (based on ADC maps only).

The third finding is that though radiomic signature contains the delta-volume, the prediction is better than the one based on delta-volume only (AUC 0.93 vs. 0.73), meaning that the radiomic signature provides larger volume-independent information compared to the volume only. Moreover, early prediction of IC response with the radiomics signature is better than radiological prediction based on the RECIST after the first cycle and performs better than a model based only on delta volume.

Despite not being the first study to use delta radiomics, the results found in this study are consistent with the literature. As a matter of fact, delta-radiomics has been previously used for various applications, in various districts and with various imaging techniques, and all those studies agree on the added value of delta-radiomics compared to the traditional radiomics. In [23], it was shown how the difference of radiomic features between two CT screenings improved early detection of cancer compared to using just the one in the first screening (classification AUC 0.82 vs. 0.77). In [24], T2w features were used to obtain a radiomic signature in rectal cancer that could be used as a prognostic factor for survival that is independent on other clinical features. In [25], delta-radiomics from T1w and T2w images was used to successfully classify good vs. bad responses to neoadjuvant chemotherapy in patients with soft tissue sarcoma, achieving an accuracy of 74.6%. Gao et al. [26] found an improvement in delta-radiomics from ADC compared with a simple difference in mean ADC (AUC 0.91 vs. 0.74). The fact that the current and the previous studies agree on the usefulness of delta radiomics in different districts suggests that the results can be generalized. Our study differs from previous studies in terms of the different types of cancer (sinonasal), the rarity of the disease, the use of ADC (which was used only in [26]) and the clinical outcome (response to IC).

From a clinical perspective, the prediction of IC treatment response could be of great importance in the multimodal approach to SNCs, even if obtained after the first cycle of therapy. On one hand, this could maximize the effectiveness of IC in patients showing an early benefit and on the other, avoid unnecessary toxicities in patients who should not achieve any response. A similar approach has already been used in laryngeal cancer, where Urba et al. [27] showed the feasibility of an appropriate selection of patients for organ preservation approaches by evaluating the response after just one cycle of IC. The authors evaluated the clinical response with direct laryngoscopy under anesthesia through bi-dimensional measurements of the primary tumor, while no radiological imaging was performed. Bioselection after a single cycle of chemotherapy is subsequently confirmed as a feasible strategy for treating laryngeal cancer. In a propensity score model, both disease-specific and overall survival were significantly improved in the group of patients receiving a single cycle neoadjuvant therapy as selection for further treatment compared to the group of patients directly admitted to definitive treatment with radiochemotherapy [28]. This approach needs to be exploited also in sinonasal cancers, as this disease has a strong prognostic factor for response to IC, in common with laryngeal cancer.

The main limitation of this study was the heterogeneity of image acquisition parameters, as the study was multicentric. However, in a recent study [18], radiomics features that are stable to various acquisition parameters were identified and used in this study; thus the results should not be biased because of acquisition parameter heterogeneity. Moreover, because SNC is a rare tumor, the number of cases was limited compared to other radiomics studies. However, the database was divided into training, validation, and test sets to produce robust results.

Another major limitation is related to the low number of patients compared with the high number of features, which tends to increase the probability of overfitting. This aspect is common in rare tumors. In fact [25,26], who worked on sarcopenia, another rare pathology, had to deal with small datasets as well (65 and 30 patients, respectively). It should be noted that sinonasal cancers are rare diseases, thus underlining the importance of the results obtained in 50 patients, and in the context of a retrospective clinical trial, with locally advanced, epithelial cancers. In this study, we attempted to perform multiple train-validation and test splits to ensure the absence of overfitting in our model; however the low number of patients translated into a low number of patients in the validation and test sets, especially for ADC, which increases the variability of the quality metrics, as can be noticed by the confidence intervals observed in Table 2 and Table 3. Future development is, of course, the enrollment of an independent validation set to further assess the quality of our findings.

Finally, another limitation of the study is the fact that, in order to increase the sample size of the dataset, we merged multiple histological subtypes, even if all had epithelial characteristics. Although the histological subgroups considered are similar among each other, it is possible that the different histology may have influenced the RECIST response (as shown in Table 1), and this influence has not been accounted for in any way by the radiomic model. This is because, given the low number of patients, it was not possible to perform a subsite-specific analysis or to develop and validate a multivariate clinical-radiomic model. This, of course, remains a possible future development of this study.

## 5. Conclusions

In this study, we showed that predictive models based on delta radiomics, in particular, including ADC-based features, can be used to predict the response to IC in sinonasal cancer. We have shown that delta-radiomics performs better than baseline-radiomics in predicting response, when using T2w images and ADC maps, which is a confirmation of previous results obtained in other cancers. The results of this study may be a first step to further increase the research on delta-radiomics for the prediction of treatment response in sinonasal cancer. The added value for the clinical management of such patients is the possibility of refining treatment strategies immediately after the first cycle of chemotherapy, thus allowing the maximization of the response to systemic treatments in chemosensitive diseases and to directly proceed to locoregional treatments in chemoresistant cancers. Moreover, this approach could help identify a subset of patients where it is possible to explore new therapeutic strategies, as they are resistant to standard antineoplastic drugs.

## Figures and Tables

**Table 1:** Patient and disease characteristics divided by response group: responders (partial and complete responses) vs. non-responders (stable disease or progression).

Characteristics	Responders	Non-Responders	*p*-Value
Male:Female	21:4	20:5	0.71
T-stage			0.357
T3	2	1	
T4a	12	8	
T4b	11	16	
Clinical trial			0.024
Study 1	17	9	
Study 2	8	16	
Histotype			0.091
SCC	5	7	
SNUC	16	7	
SNEC, ONB	4	7	
ITAC	0	4	
Imaging			
T1-weighted	23	25	0.977
T2-weighted	24	25	
ADC maps	13	15	

SCC: squamous cell cancer; SNUC: sinonasal undifferentiated cancer; SNEC: sinonasal neuroendocrine cancer; ONB: olfactory neuroblastoma; ITAC: intestinal-type adenocarcinoma. Association is evaluated with the *p*-value of a chi-squared test (*p* < 0.05 is considered statistically significant).

**Table 2:** Performance of the predictive mono-modality models built using delta radiomics between the first imaging after one cycle of IC and baseline in the validation and test sets, as well as performance of the early radiological evaluation.

Characteristics	Accuracy	TPR	TNR
Validation set (radiomics)			
T1w	0.82 ± 0.10	0.78 ± 0.16	0.86 ± 0.15
T2w	0.79 ± 0.10	0.79 ± 0.16	0.80 ± 0.15
ADC	0.89 ± 0.13	0.89 ± 0.19	0.90 ± 0.18
Test set (radiomics)			
T1w	0.80 ± 0.16	0.73 ± 0.28	0.86 ± 0.21
T2w	0.78 ± 0.17	0.75 ± 0.26	0.80 ± 0.25
ADC	0.87 ± 0.13	0.86 ± 0.19	0.89 ± 0.20
Radiological evaluation	0.78	0.6	0.96
Volume	0.72 ± 0.10	0.56 ± 0.16	0.89 ± 0.12

**Table 3:** Mean area under the ROC Curve (AUC) obtained in the test set. For each value of AUC, the corresponding 95% confidence interval is reported in brackets.

Characteristics	AUC Delta Radiomics Model	AUC Baseline Model
Mono-modality		
T1w	0.79 (0.65–0.88)	0.69 (0.55–0.81)
T2w	0.76 (0.62–0.87)	0.54 (0.51–0.78)
ADC	0.93 (0.75–1)	0.79 (0.63–0.91)
Fused signatures		
T1w + T2w	0.83 (0.70–0.92)	0.75 (0.58–0.85)
T1w + ADC	0.88 (0.74–0.95)	0.77 (0.63–0.89)
T2w + ADC	0.85 (0.73–0.95)	0.78 (0.62–0.88)
T1w + T2w + ADC	0.89 (0.75–0.95)	0.83 (0.70–0.93)

## Data Availability

The data presented in this study are available upon request from the corresponding author. The data are not publicly available because of restrictions owing to privacy issues.

## Supplementary Materials

The following supporting information can be downloaded at: https://www.mdpi.com/article/10.3390/jimaging8020046/s1, Figure S1: T1- and T2-weighted images and ADC map for one patient at baseline (A–C) and after three weeks after starting IC therapy (D–F); Table S1: Scanners and acquisition parameters used to acquire the magnetic resonance images.
