# Supplementary material for: Refining Tumor Treatment in Sinonasal Cancer Using Delta Radiomics of Multi-Parametric MRI after the First Cycle of Induction Chemotherapy
Source: J Imaging. 2022 Feb 15;8(2):46. doi: 10.3390/jimaging8020046 (PMC8877083; doi:10.3390/jimaging8020046)
Supplement: Supplementary file 1 [file jimaging-08-00046-s001.zip › jimaging-1535720-supplementary.pdf]

Supplementary Materials—Refining Tumor Treatment in Sinonasal Cancer Using Delta Radiomics of Multi-Parametric MRI after the First Cycle of Induction Chemotherapy

Valentina D. A. Corino *, Marco Bologna, Giuseppina Calareso, Carlo Resteghini, Silvana Sdao, Ester Orlandi, Lisa Licitra, Luca Mainardi and Paolo Bossi

Table S1. Scanners and acquisition parameters used to acquire the magnetic resonance images.

**Scanner**	- GE Discovery MR 750 3T (1 patient)<br>- Philipps Achieva 1.5T (3 patients)<br>- Siemens Magnetom Aera 1.5T (2 patient)<br>- Siemens Magnetom Avanto 1.5T (33 patients)<br>- Siemens Magnetom Avanto Fit 1.5T (10 patients)<br>- Toshiba MRT200PP3 (1 patient)
**Pulse sequence**	- Turbo spin-echo (T1-weighted and T2-weighted)<br>- Echo planar imaging (DWI)
**TR**	- T1-weighted (525.99 ± 66.29 ms, range 405–720 ms)<br>- T2-weighted (4.81 ± 1.42 s, range 2.65–8.81 s)<br>- DWI (5.19 ± 1.26 s, range 2.55–7.4 s)
**TE**	- T1-weighted (11.58 ± 1.27 ms, range 5.93–15 ms)<br>- T2-weighted (108.08 ± 7.66 ms, range 75–123 ms)<br>- DWI (76.11 ± 15.51 ms, range 52.5–93 ms)
**Diffusion b-values sequences**	- 0/1000 s/mm^2 (3 patients)<br>- 0/50/100/500/750/1000 s/mm^2 (25 patients)<br>- 0/10/20/50/70/150/200/500/1000 s/mm^2 (6 patients)
**Slice thickness**	- T1-weighted (3.15 ± 0.48 mm, range 3–5 mm)<br>- T2-weighted (3.12 ± 0.40 mm, range 3–5 mm)<br>- DWI (3.13 ± 0.45 mm, range 3–5 mm)
**Pixel spacing**	- T1-weighted (0.60 ± 0.10 mm, range 0.25–0.9 mm)<br>- T2-weighted (0.50 ± 0.10 mm, range 0.29–0.69 mm)<br>- DWI (1.58 ± 0.41 mm, range 0.94–2.18 mm)

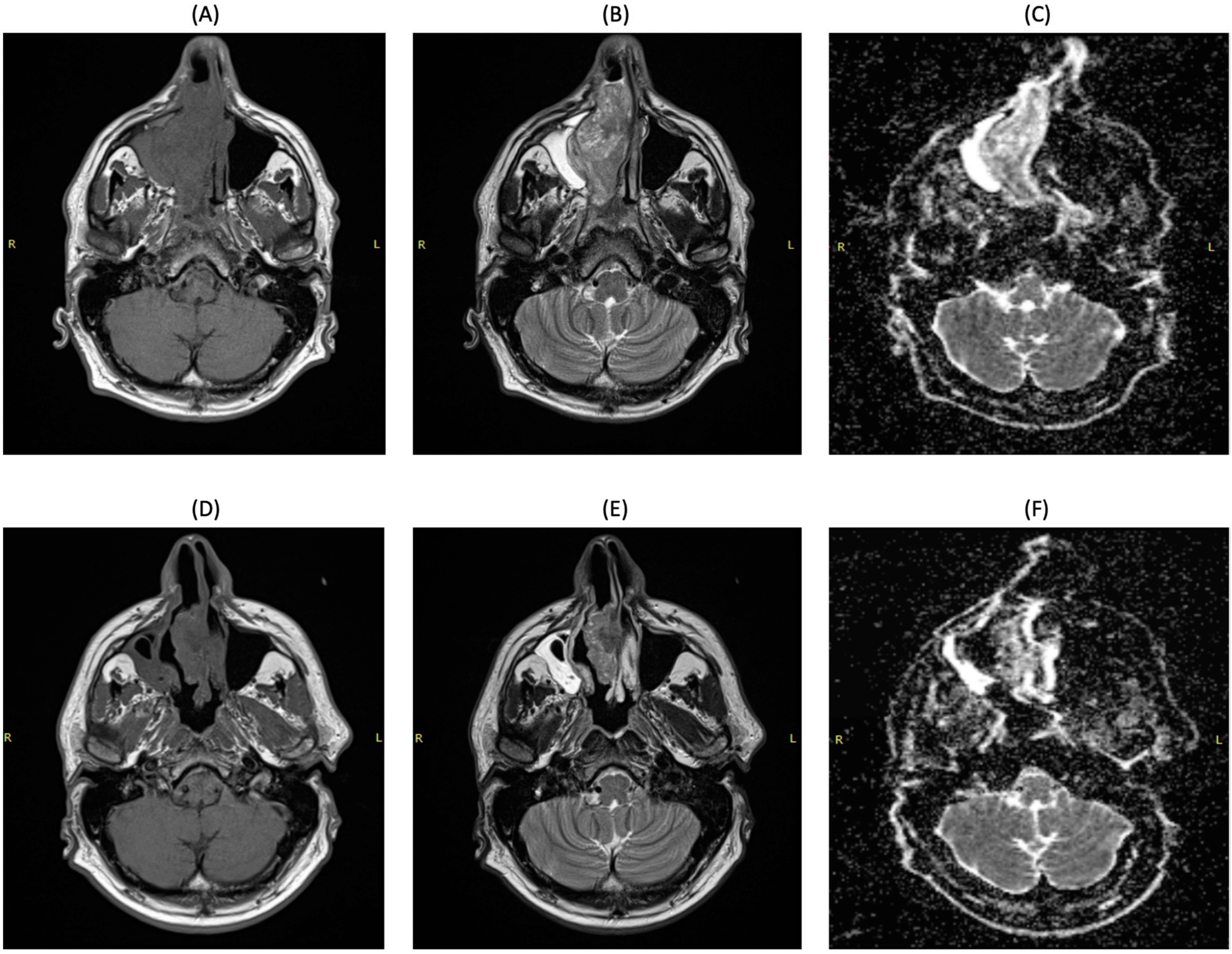

Figure S1. T1- and T2-weighted images and ADC map for one patient at baseline (**A**) – (**B**) – (**C**) and after three weeks after starting IC therapy (**D**) – (**E**) – (**F**).
